# Early non-invasive cardiac output monitoring in hemodynamically unstable intensive care patients: A multi-center randomized controlled trial

**DOI:** 10.1186/cc10273

**Published:** 2011-06-15

**Authors:** Jukka Takala, Esko Ruokonen, Jyrki J Tenhunen, Ilkka Parviainen, Stephan M Jakob

**Affiliations:** 1 Department of Intensive Care Medicine, Bern University Hospital (Inselspital), and University of Bern, Freiburgstrasse, CH-3010 Bern, Switzerland; 2 Department of Anesthesiology and Intensive Care, Kuopio University Hospital, P.O. Box 1777, 70211 Kuopio, Finland; 3 Critical Care Medicine Research Group, Department of Intensive Care Medicine, Tampere University Hospital, Teiskontie 35, 33521 Tampere, Finland

## Abstract

**Introduction:**

Acute hemodynamic instability increases morbidity and mortality. We investigated whether early non-invasive cardiac output monitoring enhances hemodynamic stabilization and improves outcome.

**Methods:**

A multicenter, randomized controlled trial was conducted in three European university hospital intensive care units in 2006 and 2007. A total of 388 hemodynamically unstable patients identified during their first six hours in the intensive care unit (ICU) were randomized to receive either non-invasive cardiac output monitoring for 24 hrs (minimally invasive cardiac output/MICO group; n = 201) or usual care (control group; n = 187). The main outcome measure was the proportion of patients achieving hemodynamic stability within six hours of starting the study.

**Results:**

The number of hemodynamic instability criteria at baseline (MICO group mean 2.0 (SD 1.0), control group 1.8 (1.0); *P *= .06) and severity of illness (SAPS II score; MICO group 48 (18), control group 48 (15); *P *= .86)) were similar. At 6 hrs, 45 patients (22%) in the MICO group and 52 patients (28%) in the control group were hemodynamically stable (mean difference 5%; 95% confidence interval of the difference -3 to 14%; *P *= .24). Hemodynamic support with fluids and vasoactive drugs, and pulmonary artery catheter use (MICO group: 19%, control group: 26%; *P *= .11) were similar in the two groups. The median length of ICU stay was 2.0 (interquartile range 1.2 to 4.6) days in the MICO group and 2.5 (1.1 to 5.0) days in the control group (*P *= .38). The hospital mortality was 26% in the MICO group and 21% in the control group (*P *= .34).

**Conclusions:**

Minimally-invasive cardiac output monitoring added to usual care does not facilitate early hemodynamic stabilization in the ICU, nor does it alter the hemodynamic support or outcome. Our results emphasize the need to evaluate technologies used to measure stroke volume and cardiac output--especially their impact on the process of care--before any large-scale outcome studies are attempted.

**Trial Registration:**

The study was registered at ClinicalTrials.gov (Clinical Trials identifier NCT00354211)

## Introduction

Hemodynamic instability early during intensive care increases the risk of morbidity and mortality. Several small studies have used pre-emptive or early hemodynamic support protocols in septic and postoperative patients to improve outcome [1-10]. In contrast, similar protocols applied later have no beneficial effect and may even worsen the outcome [11-13]. It is conceivable that reducing the delay in stabilizing hemodynamics has the potential for improving the subsequent clinical course.

Large-scale use of therapeutic protocols for early intervention has been hampered by logistic and conceptual problems. Installing invasive hemodynamic monitoring is labor-intensive per se. Use of hemodynamic management protocols assumes that appropriate goals and interventions are known. Traditional invasive hemodynamic monitoring using a pulmonary artery catheter has also been questioned [14-16].

Recently, less invasive techniques have been introduced for monitoring cardiac output, stroke volume or their surrogates [17,18]. An arterial pressure waveform-based method allows continuous estimation of cardiac output without calibration [19-21] and facilitates rapid installation of minimally invasive cardiac output (MICO) measurement in patients with an arterial line. The MICO should reflect trends in cardiac output, even if the absolute values may not be accurate.

We hypothesized that continuous monitoring of cardiac output would enhance hemodynamic stabilization in patients with unstable hemodynamics at or within six hours of admission to the intensive care unit (ICU). If this were true, reduced organ dysfunction and ICU resource use and improved outcome could be expected.

## Materials and methods

This was a multicenter, international, randomized controlled trial (Clinical Trials identifier NCT00354211; ClinicalTrials.gov). Three surgical-medical university hospital ICUs (one from Switzerland, two from Finland) participated. All units are intensivist-led closed units with 24-hour-a-day/7-day-a-week coverage by physicians with duties only in the ICU.

All emergency admissions to the ICU were screened. Patients with aortic regurgitation, with an intra-aortic balloon pump or with a pulmonary artery catheter on admission to ICU were excluded. All other patients at least 18 years of age, hemodynamically unstable at or within six hours of ICU admission, and with an arterial line, were eligible for inclusion. Since no additional interventions or deviations from usual care were included, the Ethics Committees of the hospitals (Finnish centers) and the Canton of Bern (Swiss center) approved deferred written informed consent (from the patient or the family; two centers) or deferred written informed consent and prior to study inclusion a statement of no objection to the study from an independent physician (one center).

Hemodynamic instability was defined as the presence of any of the following five criteria: 1) clinically relevant hypotension (systolic arterial pressure < 90 mmHg, acute symptomatic decrease of blood pressure, suspicion of cerebral hypoperfusion, or acute reduction in urinary output related to blood pressure decrease; 2) clinical signs of hypovolemia with or without hypotension (peripheral vasoconstriction, decreased venous filling); 3) oliguria (excluding patients with established oliguric renal failure), with diuresis < 0.5 ml/kg estimated body weight/hour; 4) elevated blood lactate (> 50% above upper normal limit) and clinical suspicion of hypoperfusion; 5) acute alteration of mental status related to hemodynamic alterations.

### Randomization and masking

The patients were randomized using fully opaque, sequentially numbered sealed envelopes and stratification by center to either have the MICO device (Flow-Trac^®^, software version 1.07, Edwards Lifesciences, Irvine, CA, USA) connected to their arterial pressure measurement system (MICO group) or to serve as controls (control group). The MICO device was installed by the research staff once an arterial line was available.

In both patient groups, treatment guidelines were made available but not enforced (see Additional file 1/Hemodynamic guidelines). Data were collected for standard demographics, simplified acute physiology score (SAPS) II [22], sequential organ failure assessment (SOFA) score [23] at admission, the primary cause of ICU admission [24], and the presence of verified or suspected infection at admission. Hemodynamic data and the five criteria of hemodynamic instability were recorded hourly for 24 hrs, or to discharge if before 24 hrs. Fluids given for volume resuscitation (total amount, type of fluids and their timing), vasoactive drugs, and use of pulmonary artery catheter were recorded. In the MICO group, the MICO device was used for the duration of hemodynamic data collection. Predicted mortalities were calculated using the SAPS II coefficients [22].

### Outcome criteria

The primary outcome variable was the proportion of patients achieving hemodynamic stability within six hours of starting the study. ICU and hospital mortality were considered as secondary outcomes. Hospital outcome was defined as outcome from the last of consecutive hospitalizations (that is, discharges to other hospitals) before discharge home or to rehabilitation. ICU outcome was defined from the last ICU stay. ICU mortality was separately analyzed post hoc for early deaths occurring during the first 24 hrs. Further secondary outcomes included total time required for hemodynamic stabilization, pulmonary artery catheter use, ICU resource use during the primary ICU admission using the simplified therapeutic intervention scoring system [25] (TISS score), ICU readmissions, and length of stay at the study institution (ICU and hospital).

### Statistical analysis

(see Additional file 1 for details on statistical analysis)

The primary hypothesis was that the use of MICO increases the proportion of patients reaching hemodynamic stability at six hours. We assumed that 50% of the patients from the control group would be stable at six hours. To detect a difference of 15% in the proportion of patients stable at six hours with a power of 90% at a 0.05 significance level, the minimum required sample size was 390, with 195 in each group, with an assumption of 5% loss of complete monitoring.

The intention-to-treat (ITT) population consisted of all patients randomized. Patients in whom hemodynamic assessments per protocol were not available were excluded from the per-protocol analysis. The primary outcome analysis for the proportion of patients stable at six hours was done in the ITT population using the chi-square test. Since patients could again become unstable after initial stabilization, time needed to reach hemodynamic stability within the first 6 hrs and 24 hrs was further analyzed post hoc by defining the time point after which none of the instability criteria were present or reappeared, and by constructing separate Kaplan-Meier curves for each time period and using the Breslow test for between-group differences. Between-group comparisons of other variables were done in the per-protocol population. The unpaired t test or the Mann-Whitney U-test was used for continuous variables and the chi-square or Fisher's exact test for proportions. Further analyses of group and study center interactions were done with analysis of variance. Changes from baseline to 6 hrs and 24 hrs in stroke volume, cardiac output and stroke volume variation in the MICO group were analyzed using repeated measures analysis of variance and paired t tests with Bonferroni correction. Logistic regression was used to detect variables associated with hospital outcome. A two-tailed *P-*value less than 0.05 was considered significant.

The data are shown as mean (SD) or median (25^th ^to 75^th ^percentile; for not normally distributed variables according to the Kolmogorov-Smirnov test), unless specified otherwise. The statistical analyses were done with SPSS (version 15.0, SPSS Inc., Chicago, IL, USA) and GraphPad InStat (version 3.05; GraphPad Software, San Diego, CA, USA).

## Results

**Figure 1:**
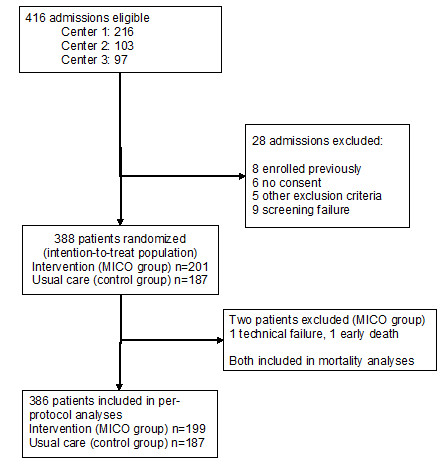
**Study flowchart**. MICO: minimally invasive cardiac output.

Of the 416 eligible patients, 388 patients were included in the intention-to-treat (ITT) analysis and 386 in the per-protocol analysis (Figure 1). The groups were comparable in terms of demographics (except a slightly younger age in the MICO group and greater weight in the control group; Table 1), type of admission (surgical vs. medical), main admission diagnosis categories, presence of infection at admission, baseline hemodynamics, severity of illness (Table 1), and presence of organ dysfunctions at baseline (see Additional file 1/Table 1). Also, the time spent in the hospital before admission to the ICU and the time from ICU admission to arterial line insertion and study baseline were comparable (Table 1). Sixty-one percent of patients were admitted to the ICU within 24 hrs of hospital admission, 42% within six hours, and 20% within one hour.

**Table 1:** Demographics, time intervals relevant to intensive care and study admission, and baseline hemodynamics

	MICO N = 199	Control N = 187	*P-*value
Age; years	59 (18)	64 (16)	0.05
Gender; n; female/male	67/132	62/125	1.00
Height; cm	171 (9)	170 (10)	0.25
Weight; kg	81 (19)	78 (18)	0.06
SAPS II	48 (18)	48 (15)	0.86
Predicted risk of death; %	43 (29)	43 (26)	0.85
Patient with > 50% risk; n	87	72	0.30
Infection, confirmed; %	27	23	0.29
Infection, suspected or confirmed; %	51	49	0.69
Surgical admission; %	32	27	0.37
Hospital admission to ICU admission;	13	13	0.49
hours	(2 to 54)	(1 to 93)	
ICU admission to arterial line insertion; minutes	20 (6 to 45)	16 (5 to 42)	0.13
Arterial line insertion to baseline;	64	45	0.12
minutes	(23-144)	(17-116)	
Systolic blood pressure (mmHg)	103 (26)	107 (27)	0.16
Diastolic blood pressure (mmHg)	53 (15)	53 (12)	0.95
Heart rate (beats/min)	97 (24)	95(25)	0.40
APACHE III diagnostic groups (%)			0.56
Medical admission			
Cardiovascular	17.1	23.0	0.16
Respiratory	14.1	18.2	0.33
Gastrointestinal	10.1	8.6	0.73
Sepsis	14.6	11.2	0.37
Other medical	12.6	11.8	0.88
Surgical admission			
Cardiovascular	5.5	7.5	0.54
Gastrointestinal	13.1	8.6	0.19
Trauma	4.5	4.8	1.00
Other surgical	8.5	6.4	0.45

Values are mean (SD), median (25^th to ^75^th ^percentile), ratio, or percentage, as indicated.

APACHE, acute physiology and chronic health evaluation; MICO, minimally invasive cardiac output; SAPS II, simplified acute physiology score II.

At baseline, clinical signs of hypovolemia and clinically relevant hypotension were the most frequent criteria of hemodynamic instability, with no significant differences between the groups (Table 2). The number of patients with at least three criteria of hemodynamic instability was higher in the MICO group (*P *= .05; Table 2).

**Table 2:** Presence of criteria for hemodynamic instability at baseline

	MICO n = 199	Control n = 187	*P-*value
Clinically relevant hypotension; n (%)	109 (55)	87 (47)	.13
Clinical signs of hypovolemia; n (%)	134 (67)	124 (66)	.91
Oliguria; n (%)	71 (36)	50 (27)	.06
Elevated blood lactate; n (%)	59 (30)	48 (26)	.43
Acute changes in mental status related to hemodynamics; n (%)	23 (12)	26 (14)	.54
Number of instability criteria; mean (SD)	2.0 (1.0)	1.8 (1.0)	.06
Number of patients with at least three criteria; n (%)	60 (30)	40 (21)	.05

MICO, minimally invasive cardiac output; SD, standard deviation.

There were no significant differences between the study groups in volumes or types of fluids received for hemodynamic support in the first six hrs (Table 3). The MICO group received slightly more colloids than the control group after 6 hrs (*P *= .01) and for the whole 24 hrs (*P *= .01). There were no differences in the frequency or type of inotropic or vasopressor drugs given, but vasodilators were used more frequently in the control group (*P *= .02; Table 3). A pulmonary artery catheter was inserted in 19% of the patients in the MICO group and in 26% in the control group (*P *= .11).

**Table 3:** Hemodynamic support with fluids and vasoactive drugs

	MICO n = 199	Control n = 187	*P-*value
Volume replacement; mL/kg			
Total			
0 to 6 hrs	11.4 (5.0 to 20.9)	9.1 (2.7 to 19.5)	.22
7 to 24 hrs	14.2 (5.6 to 28.2)	13.2 (5.4 to 29.4)	.76
0 to 24 hrs	28.6 (16.3 to 45.8)	24.9 (15.0 to 48.0)	.40
Colloids			
0 to 6 hrs	5.8 (0.0 to 12.1)	4.9 (0.0 to 9.7)	.33
7 to 24 hrs	6.7(1.9 to 14.8)	5.7 (0.0 to 13.0)	.06
0 to 24 hrs	14.9 (6.7 to 22.1)	11.0 (5.6 to 23.3)	.09
Crystalloids			
0 to 6 hrs	0.0 (0.0 to 8.0)	0.0 (0.0 to 7.0)	.54
7 to 24 hrs	0.0 (0.0 to 11.0)	0.0 (0.0 to 13.0)	.58
0 to 24 hrs	6.0 (0.0 to 22.0)	6.0 (0.0 to 20.0)	.81
Blood products			
0 to 6 hrs	0.0 (0.0 to 4.0)	0.0 (0.0 to 3.0)	.76
7 to 24 hrs	0.0 (0.0 to 0.0)	0.0 (0.0 to 0.0)	.62
0 to 24 hrs	0.0 (0.0 to 6.0)	0.0 (0.0 to 7.0)	.64
Any vasoactive drug	150 (75)	150 (80)	.27
dobutamine	44 (22)	45 (24)	.72
noradrenaline	115 (58)	103 (55)	.61
adrenaline	19 (10)	14 (7)	.59
vasodilator	26 (13)	42 (22)	.02
beta-blocking agents	6 (3)	14 (7)	.07
other anti-arrhythmic drugs	14 (7)	23 (12)	.09

Fluids given for basal daily needs of water and electrolytes (maintenance fluids) excluded. Values are median (interquartile range) for fluids and number of patients (percentage) receiving vasoactive drugs.

MICO, minimally invasive cardiac output.

At six hours, 45 patients (22%) in the MICO group and 52 patients (28%) in the control group were hemodynamically stable (*P *= .24). In each group, 19 patients (10%) became stable before 6 hrs and remained stable thereafter (see Additional file 1/Table 2); 34 patients in each group were stable at each time point (6 hrs and 24 hrs). The Kaplan-Meier curves for reaching hemodynamic stability (the time point after which none of the instability criteria were present or reappeared; see Additional file 1/Figure 1) for 6 hrs showed no difference between the groups (*P *= .250). The presence of individual criteria of hemodynamic instability at baseline and mechanical ventilation at baseline were compared between stable and unstable patients at 6 hrs, and variables with differences with a *P-v*alue less than .10 were included in logistic regression analysis. Only an acute symptomatic decrease in blood pressure at baseline (*P *= .050) and increased blood lactate at baseline (*P *= .002) were significant predictors of instability at six hours (for additional details, see Additional file 1/Results).

In the MICO group, stroke volume increased marginally [baseline: 65 mL (20 mL), 6 hrs: 65 mL (20 mL), 24 hrs: 69 mL (20 mL); *P *= .002] and stroke volume variation decreased slightly over time (baseline: 16% (12%); 6 hrs: 15% (10%); 24 hrs: 14% (10%): *P *= .05), whereas cardiac output did not change significantly (baseline: 5.9 (2.0) L/minute, 6 hrs: 5.9 (1.8) L/minute; 24 hrs: 6.1 (1.8) L/minute: *P *= .23].

Neither the ICU mortality nor the hospital mortality was significantly different between the groups (ICU mortality MICO 18%, control 12%, *P *= .122, relative risk = 1.497

95% confidence interval 0.925 to 2.42; hospital mortality MICO 26%, control 21%, *P *= .34, risk = 1.209, 95% confidence interval .843 to 1.735). There were more deaths from randomization until 24 hrs after end of study monitoring in the MICO group (MICO n = 16 vs. control n = 4, *P *= .011). All but one of these deaths (in the MICO group) occurred after withdrawal of treatment due to poor prognosis of the underlying condition (for details, see Additional file 1/Table 3).

**Figure 2:**
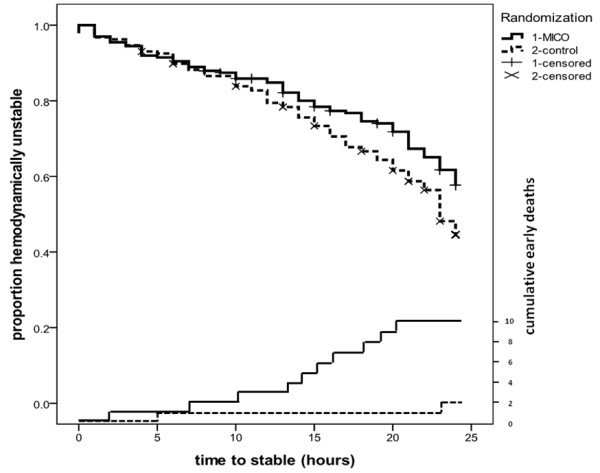
**Hemodynamic stability (*P *= 0.033) and cumulative occurrence of deaths within the first 24 hrs**. MICO: minimally invasive cardiac output.

The 24-hr Kaplan-Meier curves (Figure 2) indicated that patients in the control group were more likely to reach hemodynamic stability (*P *= .033). After excluding the early deaths, the difference was not significant (*P *= .11). When stratified according to number of instability criteria at baseline (≥ 3 vs. < 3 criteria), the difference between MICO and control groups was not significant (*P *= .79 for ≥ 3 criteria; *P *= .12 for < 3 criteria).

Differences between survivors and non-survivors were tested for study group, presence of each of the individual criteria of hemodynamic instability at baseline and 6 hrs, mechanical ventilation at baseline and 6 hrs, stability at 6 hrs and 24 hrs, and time to achieve hemodynamic stability. Those with a *P-*value less than .10 were included in logistic regression analysis. Only oliguria (*P *= .000) and increased lactate (*P *= .01) at baseline were significant predictors of hospital mortality (for additional details, see Additional file 1/Results and Additional file 1/Tables 4-8).

Resource utilization was similar in the two groups. The TISS score for the first 24 hrs in the ICU was 32 (8) in the MICO group and 32 (9) in the control group (*P *= .79). The sum of TISS scores for the whole ICU stay was 172 (215) in the MICO group and 174 (194) in the control group (*P *= .93). The median length of stay in the ICU was 2.0 (1.2 to 4.6) days in the MICO group and 2.5 (1.1 to 5.0) days in the control group (*P *= .38). The duration from ICU admission to hospital discharge was 11.0 (5.6 to 18.3) days in the MICO group and 10.4 (5.3 to 18.6) days in the control group (*P *= .78). Twenty-three patients (11%) in the MICO group and 29 (16%) in the control group were later readmitted to the ICU (*P *= .30).

## Discussion

The main finding of the study was that providing continual cardiac output and stroke volume monitoring non-invasively during treatment of hemodynamic instability did not increase the proportion of patients achieving hemodynamic stability during the early phase of treatment. The use of fluids and catecholamines was very similar in the two study groups, suggesting that the availability of additional hemodynamic information did not change the treatment as compared to standard therapy. Given the similarity of treatments received, and assuming that the severity and main characteristics of the underlying clinical problems also reflected similar pathophysiology of hemodynamic instability in both groups, it should be expected that the main clinical outcomes were not different. Our assumption that the availability of more information would help to accelerate hemodynamic stabilization is clearly not supported by the results.

Hemodynamic monitoring in intensive care has been controversial over the past decades. Use of the pulmonary artery catheter has markedly decreased due to studies suggesting that its routine use does not improve clinically relevant outcomes [14-16,26-31], and the costs associated with its use are substantial. Nevertheless, early hemodynamic instability is indisputably a risk factor for poor outcome [32-35]. Accordingly, alternative techniques for hemodynamic monitoring have been introduced and adopted for widespread clinical use despite the lack of proof of efficacy and despite the costs involved [17-21,36].

We chose to use the proportion of patients stable at six hours, an outcome reflecting the care process, as the primary outcome variable, because early treatment of hemodynamic instability has been perceived as crucial for improving outcome. If this cannot be achieved, any improvements in other outcomes are unlikely.

Our study can be criticized due to the lack of a treatment protocol combined with the use of a monitoring device - a feature that is common to most previous studies on hemodynamic monitoring, especially those on the pulmonary artery catheter [15,16]. Several single-center and small-scale studies have shown improved clinically relevant outcomes when hemodynamic monitoring has been combined with the use of protocols to guide treatment [1-10]. Protocolized care is problematic for various reasons, such as difficulties in defining the "right" intervention, acceptance in routine clinical use, compliance, and efficacy outside clinical trials. Furthermore, new monitoring devices are normally introduced into clinical practice without the use of proven protocols to achieve efficacy.

Despite the lack of protocol use in our study, almost half of the fluids were given during the first six hours, suggesting more intensive treatment during the early ICU stay. We further evaluated (data not shown) patients receiving little fluids for volume expansion during the first six hours (< 5 ml/kg); most of these had either a cardiac or respiratory (APACHE III diagnoses cardiac arrest, cardiac failure, acute myocardial infarction, bacterial or aspiration pneumonia) cause for ICU admission, which is likely to explain the restrictive volume supply, or were admitted postoperatively having already received treatment.

We expected that rapid availability of stroke volume and cardiac output at the bedside would help clinicians select interventions resulting in enhanced stabilization. There are several possible reasons why this failed. The reliability and accuracy of arterial pulse wave-based stroke volume and cardiac output technologies to track changes can be questioned [20,21,37]. Hence, the clinicians possibly did not trust the information. Indirectly, more frequent use of the pulmonary artery catheter to gain more hemodynamic data in the control group speaks against this. All participating centers had 24-hour-a-day, 7-day-a-week in-unit coverage by intensive care physicians, and all centers used invasive hemodynamic monitoring routinely. This may have facilitated the care process in both groups, and any potential improvements would be difficult to achieve. We consider this explanation unlikely, despite the substantially lower than expected hospital mortality (MICO 26% vs. 44%, control 21% vs. 43%). Despite the high physician coverage, the proportion of patients who became hemodynamically stable within 6 hrs and remained stable was low (only 10% in both groups), and less than half of the patients reached stability within 24 hrs. This was clearly less than expected in our power calculations, and would have reduced the chance of finding any difference. The lower than expected statistical power is probably not relevant, because not even a numeric trend in favor of MICO was seen for any relevant outcome. More importantly, the low rate of reaching stability suggests that the care process could have been improved. We consider that our results provide another example of the difficulties in changing clinical practice. In the future, combining such technologies with care protocols should be tested.

Patients in the control group were more likely to achieve hemodynamic stability at 24 hrs than patients in the MICO group. Despite lack of differences in achieving stability at six hours and in-hospital mortality, the possibility that stroke volume and cardiac output data delayed interventions should be considered. The similarity of the treatments, including the marginally higher use of colloids after six hours in the MICO group, speaks against this. The more frequent use of vasodilators in the control group is also unlikely to explain the difference at 24 hrs due to the small number of patients with vasodilators. Also, there were no differences between the groups in the frequency of each of the hemodynamic instability criteria in patients still in the ICU at 24 hrs (see Additional file 1/Tables 3-7). Subtle differences in the case mix are more likely to explain this result, especially the substantially higher number of patients in the MICO group who died early after discontinuation of treatment due to poor prognosis of the underlying disease. Most importantly, these early deaths in the MICO group markedly increased the proportion of patients unstable at 24 hrs. In addition, patients in the MICO group tended to have more instability criteria at baseline (*P *= .06), and more MICO patients had at least three instability criteria, suggesting more severe illness at baseline.

Our study has additional limitations. Results obtained with a specific device for stroke volume and cardiac output monitoring should not be generalized. The specific MICO technology used in the present study has been associated with problems of accuracy, especially in high cardiac output states [21,37], and newer software versions have been developed to address this [37,38]. Our results emphasize the need to evaluate these technologies - especially their impact on the process of care - before any large-scale outcome studies are attempted. The study centers had very high physician coverage, which may be rather exceptional. It is conceivable that the effect on process of care may be highly dependent on staff availability and presence.

## Conclusions

In a heterogeneous population of hemodynamically unstable critically ill patients, early, non-invasive continuous cardiac output monitoring did not shorten the time to reach hemodynamic stability, produce any outcome benefit, or reduce the amount of resources used during the ICU stay as compared to standard treatment. The similarity of treatments received in both groups suggests that the availability of cardiac output data did not change the care process. Our results do not exclude the possibility that this device, when combined with a specific care protocol or used in other circumstances or with other non-invasive continuous cardiac output monitoring technologies, could have an impact on relevant outcomes. Future trials should test whether non-invasive cardiac output monitoring combined with a treatment protocol can shorten the time to reach hemodynamic stability.

## Key messages

• Only a minority of patients (22% in the MICO group and 28% in usual care) could be stabilized in the first six hours in the ICU.

• MICO did not facilitate hemodynamic stabilization during the first 24 hrs of intensive care.

• Additional monitoring with MICO *per se *had no effect on the care process (additional monitoring, fluid and vasoactive therapy).

• MICO should be tested in combination with treatment protocols to enhance early hemodynamic stabilization in the intensive care environment.

## Abbreviations

APACHE: Acute Physiology and Chronic Health Evaluation; ITT: intention-to-treat; MICO: minimally invasive cardiac output; SAPS: simplified acute physiology score; SOFA: sequential organ failure assessment; TISS: therapeutic intervention scoring system.

## Competing interests

The study was sponsored by Edwards Lifesciences (Anaheim, CA) through research contracts with the participating institutions. Each participating center supported the study by additional departmental funds. The sponsor provided the cardiac output monitors and paid travel expenses for organizational meetings.

The Department of Intensive Care Medicine at the Bern University Hospital has, or has had in the past, research contracts with Abbott Nutrition International, B. Braun Medical AG, CSEM SA, Edwards Lifesciences Services GmbH, Kenta Biotech Ltd, Maquet Critical Care AB, Omnicare Clinical Research AG, and Orion Corporation; and research and development/consulting contracts with Edwards Lifesciences SA, Maquet Critical Care AB, and Nestlé. The money is/was paid into a departmental fund; no author receives/received any personal financial gain.

The Department of Anesthesiology and Intensive Care at Kuopio University Hospital has research contracts with Orion Corporation, Eli Lilly and Arstellas. The money is paid to the institution.

The Department of Anesthesiology and Intensive Care at Tampere University Hospital received an unrestricted grant from Eli Lilly for an international scientific symposium in 2009. The money was paid to the institution.

Jyrki Tenhunen received reimbursement for travel expenses in connection with membership on the General Electric advisory board until 2008 and reimbursement for travel to board meetings of the Dexmedetomidine advisory Board/Orion Ltd. until 2009. He received money from Eli Lilly for consulting related to co-authorship on a phase II trial.

## Authors' contributions

JT conceived the study idea, designed the study, was the principal investigator and wrote the final version of the manuscript. ER and SMJ designed the study, were country coordinators and wrote the final version of the manuscript. JJT, IP and SMJ were investigators responsible for the individual centers. All authors provided comments on the drafts of the report. All authors have seen and approved the final version. The investigators had full access to all original data, performed data analyses (JT and SMJ), and wrote the manuscript independently of the study sponsor. The corresponding author had final responsibility for the decision to submit the paper for publication. The corresponding author (Jukka Takala) had full access to all the data in the study and takes responsibility for the integrity of the data and the accuracy of the data analysis.

## Supplementary Material

Additional file 1 **This manuscript is accompanied by an additional file containing the following: **1. Hemodynamic guidelines MICO group/controls; 2. Additional details on statistical analysis; 3. Figure 1. Kaplan-Meier curves for achieving hemodynamic stability within the first 6 hrs; 4. Table 1. Organ dysfunction at baseline according to the SOFA score*; 5. Table 2. Patterns of achieving and maintaining hemodynamic stability and related outcome; 6. Table 3. Details of the 20 early deaths, that is, those occurring from randomization until 24 hours after end of study hemodynamic monitoring; 7. Table 4. Clinically relevant hypotension; 8. Table 5. Clinical signs of hypovolemia with or without hypotension; 9. Table 6. Oliguria (excluding patients with established oliguric renal failure), with diuresis < 0.5 ml/kg estimated body weight/hr; 10. Table 7. Elevated blood lactate (> 50% above upper normal limit) and clinical suspicion of hypoperfusion; 11. Table 8. Acute alteration of mental status related to hemodynamic alterations; 12. Additional results Click here for file
