# Supplementary material for: Early non-invasive cardiac output monitoring in hemodynamically unstable intensive care patients: A multi-center randomized controlled trial
Source: Crit Care. 2011 Jun 15;15(3):R148. doi: 10.1186/cc10273 (PMC3219022; doi:10.1186/cc10273)
Supplement: Additional file 1 — This manuscript is accompanied by an additional file containing the following: 1. Hemodynamic guidelines MICO group/controls; 2. Additional details on statistical analysis; 3. Figure 1. Kaplan-Meier curves for achieving hemodynamic stability within the first 6 hrs; 4. Table 1. Organ dysfunction at baseline according to the SOFA score*; 5. Table 2. Patterns of achieving and maintaining hemodynamic stability and related outcome; 6. Table 3. Details of the 20 early deaths, that is, those occurring from randomization until 24 hours after end of study hemodynamic monitoring; 7. Table 4. Clinically relevant hypotension; 8. Table 5. Clinical signs of hypovolemia with or without hypotension; 9. Table 6. Oliguria (excluding patients with established oliguric renal failure), with diuresis < 0.5 ml/kg estimated body weight/hr; 10. Table 7. Elevated blood lactate (> 50% above upper normal limit) and clinical suspicion of hypoperfusion; 11. Table 8. Acute alteration of mental status related to hemodynamic alterations; 12. Additional results [file cc10273-S1.DOC]

**Additional file 1**

**Takala J et al:**

**Early non-invasive cardiac output monitoring in hemodynamically unstable intensive care patients: a multi-center randomized controlled trial**

This file contains:

1. Hemodynamic guidelines MICO group/ controls

2. Additional details on statistical analysis

3. Figure 1 Kaplan-Meier curves for achieving hemodynamic stability within the first 6 hrs

4. Table 1 Organ dysfunction at baseline according to the SOFA score*

5. Table 2 Patterns of achieving and maintaining hemodynamic stability and related outcome

6. Table 3 Details of the 20 early deaths, i.e., those occurring from randomization until 24 hours after end of study hemodynamic monitoring.

7. Table 4 Clinically relevant hypotension

8. Table 5 Clinical signs of hypovolemia with or without hypotension

9. Table 6 Oliguria (excluding patients with established oliguric renal failure), with diuresis < 0.5 ml/kg estimated body weight/hr

10. Table 7 Elevated blood lactate (> 50% above upper normal limit) and clinical suspicion of hypoperfusion

11. Table 8 Acute alteration of mental status related to hemodynamic alterations

12. Additional results

**1. Hemodynamic guidelines**

**Hemodynamic guidelines minimally invasive cardiac output** (**MICO) group**

1. Is there a problem (as defined in instability criteria)?
   1. If no, set interval for re-evaluation
   2. If yes, set hemodynamic targets
2. Check volume status:
   1. If hypovolemic, give volume challenge (define fluid and limits)
   2. If hypervolemic and blood pressure not too low, reduce preload
   3. If volume status adequate and blood pressure not too low, reduce afterload
   4. Re-evaluate
3. If blood pressure is too low
   1. Is cardiac output sufficient (MICO data)?
      1. If cardiac output not sufficient, treat a possible mechanical problem or arrhythmia; otherwise, start or increase inotropic medication
      2. Re-evaluate
      3. If cardiac output sufficient, and low vascular tone, start or increase vasopressors
      4. Re-evaluate
      5. If cardiac output sufficient and normal vascular tone, search for other than hemodynamic problems, diagnose and treat
      6. Re-evaluate

**Hemodynamic guidelines, control group**

1. Is there a problem (as defined in instability criteria)?
   1. If no, set interval for re-evaluation
   2. If yes, set hemodynamic targets
2. Check volume status:
   1. If hypovolemic, give volume challenge (define fluid and limits)
   2. If hypervolemic and blood pressure not too low, reduce preload
   3. If volume status adequate and blood pressure not too low, reduce afterload
   4. Re-evaluate
3. If blood pressure is too low
   1. Search for other than hemodynamic problems, diagnose, treat and re-evaluate
4. If blood pressure is too low, is more information on hemodynamics needed for treatment?
   1. If not, treat as considered appropriate, and re-evaluate
   2. If yes, insert a pulmonary artery catheter (consider echo)
      1. Is cardiac output sufficient?
      2. If cardiac output not sufficient, treat a possible mechanical problem or arrhythmia; otherwise, start or increase inotropic medication
      3. Re-evaluate
      4. If cardiac output sufficient, and low vascular tone, start or increase vasopressors
      5. Re-evaluate

**2. Statistics**

**Additional details on statistical analysis**

The primary hypothesis was that the continuous measurement of cardiac output increases the proportion of patients reaching hemodynamic stability at 6 hrs. We assumed that 50% of the patients from the control group would be stable at 6 hours. To detect a difference of 15% in the proportion of patients stable at 6 hours with a power of 90% at a 0.05 significance level, the minimum required sample size was 390 with 195 in each group, with an assumption of 5% loss of complete monitoring. The intention-to-treat (ITT) population consisted of all patients randomized. Patients in whom hemodynamic assessments per protocol were not available were excluded from the per-protocol analysis. The primary outcome analysis for proportion of patients stable at 6 hrs was done in the ITT population using the chi-square test. This primary analysis does not take into account the fact that after having reached the criteria of hemodynamic stability, patients may again become unstable and oscillate between being stable and unstable during the 24-hr study period. Therefore, a series of post-hoc analyses were performed. Also, it is not uncommon for the type of patients studied to require diagnostic and therapeutic interventions both within and outside the intensive care unit (ICU) during the first 24 hrs, and study data collection in those circumstances may not be logistically possible. Hence, imputation rules were established for missing data to define the time point when the patient was considered stable and to record the hemodynamics. These imputation rules included:

1. Baseline hemodynamic data were obtained as the first complete set available within the first hour of study inclusion. This was necessary to avoid, e.g., the impact of ongoing instrumentations at ICU admission.
2. For any missing hemodynamic data after baseline, the principle of last observation carried forwards was applied, if a value was available in the immediately preceding hour, until death, discharge, or end of study, whichever occurred first.
3. Patients who died, or whose treatment was discontinued while unstable (and who subsequently died within 24 hrs of the last study measurement) within the 6 hrs respective 24 hrs, were considered unstable until 6 hrs respective 24 hrs (worst ranking in the Kaplan-Meier analyses). Patients who were discharged without treatment restrictions before completing the 24 hrs of study measurements were censored at the time of last available measurement.
4. Time to reach stability was defined as the time point after which none of the instability criteria were present or reappeared; accordingly, only the data before and after any missing data point were taken into account.

In order to analyze the time pattern of reaching hemodynamic stability, separate Kaplan–Meier curves were constructed for each time period (up to 6 hrs and up to 24 hrs), with the Breslow test for between-group differences. This approach was selected since patients who were considered stable at the primary analysis end-point at 6 hrs could again become unstable later. In addition, the various scenarios of stability were separately tabulated: 1) Stable at 6 hrs, remained stable; 2) Stable at 6 hrs, not at 24 hrs (again unstable), 3) Unstable at 6 hrs, stable at 24 hrs; 4) Stable at 6 hrs, unstable later, again stable at 24 hrs; 5) Unstable at both 6 hrs and 24 hrs (remained unstable).

Between-group comparisons of other variables were done in the per-protocol population. An unpaired t-test was used for continuous variables and chi-square or Fisher’s exact test for proportions. Further analyses of group and study center interactions were done with analysis of variance. Changes from baseline to 6 hrs and 24 hrs in stroke volume, cardiac output and stroke volume variation in the minimally invasive cardiac output (MICO) group were analyzed using repeated measures analysis of variance (for patients remaining in the ICU at 24 hrs). Since a relevant number of patients died or were discharged between 6 hrs and 24 hrs, additional analyses for the MICO device values between baseline and 6 hrs and between 6 hrs and 24 hrs were done using paired t tests with Bonferroni correction for multiple comparisons. Logistic regression was used to detect variables associated with hemodynamic status at 6 hrs (stable/unstable) and hospital outcome. Variables that were different at p=0.10 level between survivors and non-survivors in univariate analysis were included in the multivariate logistic regression analysis. A two-tailed p value less than 0.05 was considered significant. The data are shown as mean (standard deviation/SD) or median (25th-75th percentile; for not normally distributed variables according to the Kolmogorov-Smirnov test), unless specified otherwise. The statistical analyses were done with SPSS (v.15.0, SPSS Inc., Chicago, IL) and GraphPad InStat (v. 3.05; GraphPad Software, San Diego, CA).

**3. Figure 1**

**Kaplan-Meier curves for achieving hemodynamic stability within the first 6 hrs**

MICO = minimally invasive cardiac output

**4. Table 1**

Organ dysfunction at baseline according to the SOFA score*

| **Organ failures at baseline**  (SOFA score) | **MICO**  n=199  median  (interquartile range) | **Control**  n=187  median  (interquartile range) | **p value** |
| --- | --- | --- | --- |
| **Cardiovascular** | 2.0 (1.0-3.0) | 2.0 (1.0-3.0) | 0.93 |
| **Respiratory** | 2.0 (1.0-3.0) | 2.0 (1.0-3.0) | 0.39 |
| **Coagulation** | 0.0 (0.0-2.0) | 0.0 (0.0-1.0) | 0.08 |
| **Hepatic** | 0.0 (0.0-0.0) | 0.0 (0.0-0.0) | 0.43 |
| **Renal** | 0.0 (0.0-1.0) | 0.0 (0.0-1.0) | 0.95 |
| **sum SOFA** | 6.0 (4.0-8.0) | 6.0 (4.0-8.0) | 0.68 |

SOFA = sequential organ failure assessment score^23^

MICO = minimally invasive cardiac output

*Excluding central nervous dysfunction due to the effect of frequent use of sedative and analgesic drugs

**5. Table 2**

Patterns of achieving and maintaining hemodynamic stability and related outcome

| **Scenario** | **MICO**  (n=199) | **Control**  (n=187) | **Total**  (n=386) |
| --- | --- | --- | --- |
|  | number of deaths / number of patients in each scenario (hospital mortality %) | | |
| **EARLY STABILIZATION**   - Stable at 6 hrs, remained stable | 0/19 (0) | 3/19 (15.8) | 3/38 (7.9) |
| **LATE STABILIZATION**   - Unstable at 6 hrs, stable at 24 hrs; - Stable at 6 hrs, unstable later, again   stable at 24 hrs | 10/60 (16.7) | 13/80 (16.3) | 23/140 (16.4) |
| **STABLE EARLY, UNSTABLE AT 24 hrs**   - Stable at 6 hrs, not at 24 hrs (again   unstable) | 4/11 (36.4) | 3/18 (16.7) | 7/29 (24.1) |
| **PERSISTENTLY UNSTABLE**   - Unstable at both 6 hrs and 24 hrs | 37/109 (33.9) | 21/70 (30.0) | 58/179 (32.4) |

MICO = minimally invasive cardiac output

The presence of each of the five criteria of hemodynamic instability at 24 hrs is shown in the following statistical analysis outputs (Tables 3-7).

Randomization Group 1 = MICO, Group 2 = control

**6. Table 3**

**Details of the 20 early deaths, i.e., those occurring from randomization until 24 hours after end of study hemodynamic monitoring.**

| **Patient number** | **Study group** | **Underlying disease (APACHE III) and cause of death** |
| --- | --- | --- |
| 1 | MICO | Operated for GI malignancy, developed postoperatively refractory septic shock, therapy withdrawn |
| 2 | MICO | Status post CPR, severe hypoxic brain injury, therapy withdrawn |
| 3 | MICO | Status post CPR, severe circulatory failure and hypoxic brain injury, therapy withdrawn |
| 4 | MICO | Other hematologic disease; newly diagnosed hematologic malignancy with massive uncontrollable pulmonary and gastrointestinal bleeding, therapy withdrawn |
| 5 | Control | Pulmonary embolism, severe circulatory failure, therapy withdrawn |
| 6 | Control | Status post CPR, severe hypoxic brain injury, discharged to die, terminal comfort care |
| 7 | Control | Other non-operative GI disease: acute pancreatitis- induced severe septic shock, therapy withdrawn |
| 8 | MICO | Status post CPR, severe mesenteric ischemia, therapy withdrawn |
| 9 | MICO | Status post CPR, acute paracetamol intoxication-induced liver failure and multiple organ failure, therapy withdrawn |
| 10 | MICO | Intra-abdominal bleeding; advanced malignancy, discharged to die, terminal comfort care |
| 11 | MICO | Status post CPR, severe cardiovascular failure, therapy withdrawn |
| 12 | MICO | Other non-operative GI disease; acute pancreatitis, died without therapy limitation |
| 13 | MICO | Peripheral arterial bypass surgery; postoperative intestinal necrosis, therapy withdrawn |
| 14 | MICO | Traumatic brain injury; multiple injury; pneumonia and septic shock; died with therapy limitation |
| 15 | Control | GI perforation; sepsis with intra-abdominal abscess, disseminated intravascular coagulation and mesenteric thrombosis, therapy withdrawn |
| 16 | MICO | Sepsis (non-urosepsis);erysipelas with septic shock, cardiac insufficiency and severe pulmonary hypertension; died with therapy limitation |
| 17 | MICO | Sepsis (non-urosepsis);pneumonia and septic shock, died with therapy limitation |
| 18 | MICO | Sepsis (non-urosepsis); septic shock, malignant lymphoma, multiorgan failure; died with therapy limitation |
| 19 | MICO | Renal disease; multiorgan failure, died after therapy withdrawn |
| 20 | MICO | Status post CPR, died with therapy limitation |
| **Patient number** | **Study group** | **Hemodynamic stability until last study measurement (completion of the 24 hrs, death, or withdrawal of therapy); time of death** |
| 1 | MICO | Unstable until 24 hrs, died 6 hrs later after therapy withdrawal |
| 2 | MICO | Unstable until 17 hrs, died l hr later after therapy withdrawal |
| 3 | MICO | Unstable at 6 hrs and at 24 hrs, temporarily stable in between; died 10 hrs later after therapy withdrawal |
| 4 | MICO | Unstable after 1 hr, died 5 hrs later after therapy withdrawal |
| 5 | Control | Unstable after 1 hr, died 3 hrs later after therapy withdrawal |
| 6 | Control | Stable at 6 hrs and at 19 hrs, thereafter discharged to die and died after 15 hrs |
| 7 | Control | Unstable until 24 hrs, died at 24 hrs after therapy withdrawal |
| 8 | MICO | Unstable until 23 hrs, died 11 hrs later after therapy withdrawal |
| 9 | MICO | Unstable until 20 hrs, died 1 hr later after therapy withdrawal |
| 10 | MICO | Unstable at 6 hrs, stable at 24 hrs, discharged to die 13 hrs after last study measurement, died 4 hrs later, i.e., 17 hrs after completing the 24-hr study period |
| 11 | MICO | Unstable until 15 hrs, died at 15 hrs after therapy withdrawal |
| 12 | MICO | Unstable until 12 hrs, died under full treatment |
| 13 | MICO | Unstable until 5 hrs, died 2 hrs later after therapy withdrawal |
| 14 | MICO | Unstable until 19 hrs, died at 19 hrs after therapy limitation |
| 15 | Control | Unstable until 13 hrs, died less than 1 hr later after therapy withdrawal |
| 16 | MICO | Unstable until 24 hrs, died with therapy limitation 9 hrs later |
| 17 | MICO | Unstable until 16 hrs, died less than 1 hr later after therapy limitation |
| 18 | MICO | Unstable until 24 hrs, died 11 hrs later after therapy limitation |
| 19 | MICO | Unstable until 24 hrs, died 21 hrs later after therapy withdrawal |
| 20 | MICO | Unstable until 19 hrs, died less than 1 hr later after therapy limitation |

MICO = minimally invasive cardiac output. APACHE = Acute Physiology and Chronic Health Evaluation. GI = gastrointestinal. CPR = cardiopulmonary resuscitation. Hrs = hours.

**7. Table 4**

**Clinically relevant hypotension**

**Randomization * hypotension cross tabulation**

|  | |  | **Hypotension** | | **Total** |
| --- | --- | --- | --- | --- | --- |
|  | |  | **0** | **1** | 0 |
| **Randomization** | 1 | Count | 123 | 42 | 165 |
|  |  | % within randomization | 74.5% | 25.5% | 100.0% |
|  |  | % within hypotension | 50.2% | 55.3% | 51.4% |
|  |  | % of total | 38.3% | 13.1% | 51.4% |
|  | 2 | Count | 122 | 34 | 156 |
|  |  | % within randomization | 78.2% | 21.8% | 100.0% |
|  |  | % within hypotension | 49.8% | 44.7% | 48.6% |
|  |  | % of total | 38.0% | 10.6% | 48.6% |
| **Total** | | Count | 245 | 76 | 321 |
|  | | % within randomization | 76.3% | 23.7% | 100.0% |
|  | | % within hypotension | 100.0% | 100.0% | 100.0% |
|  | | % of total | 76.3% | 23.7% | 100.0% |

**Chi-Square Tests**

|  | **Value** | **Df** | **Asymp. Sig. (2-sided)** | **Exact Sig. (2-sided)** | **Exact Sig. (1-sided)** |
| --- | --- | --- | --- | --- | --- |
| **Pearson Chi-Square** | .594(b) | 1 | .441 |  |  |
| **Continuity Correction(a)** | .409 | 1 | .522 |  |  |
| **Likelihood Ratio** | .595 | 1 | .440 |  |  |
| **Fisher's Exact Test** |  |  |  | .512 | .261 |
| **Linear-by-Linear Association** | .592 | 1 | .441 |  |  |
| **N of Valid Cases** | 321 |  |  |  |  |

a Computed only for a 2x2 table

b 0 cells (.0%) have expected count less than 5. The minimum expected count is 36.93.

**8. Table 5**

**Clinical signs of hypovolemia with or without hypotension**

**Randomization * hypovolemia cross tabulation**

|  | |  | **Hypovolemia** | | **Total** |
| --- | --- | --- | --- | --- | --- |
|  | |  | 0 | 1 | 0 |
| **Randomization** | 1 | Count | 126 | 39 | 165 |
|  |  | % within randomization | 76.4% | 23.6% | 100.0% |
|  |  | % within hypovolemia | 52.1% | 49.4% | 51.4% |
|  |  | % of total | 39.3% | 12.1% | 51.4% |
|  | 2 | Count | 116 | 40 | 156 |
|  |  | % within randomization | 74.4% | 25.6% | 100.0% |
|  |  | % within hypovolemia | 47.9% | 50.6% | 48.6% |
|  |  | % of total | 36.1% | 12.5% | 48.6% |
| **Total** | | Count | 242 | 79 | 321 |
|  | | % within randomization | 75.4% | 24.6% | 100.0% |
|  | | % within hypovolemia | 100.0% | 100.0% | 100.0% |
|  | | % of total | 75.4% | 24.6% | 100.0% |

**Chi-Square Tests**

|  | **Value** | **df** | **Asymp. Sig. (2-sided)** | **Exact Sig. (2-sided)** | **Exact Sig. (1-sided)** |
| --- | --- | --- | --- | --- | --- |
| **Pearson Chi-Square** | .174(b) | 1 | .677 |  |  |
| **Continuity Correction(a)** | .082 | 1 | .774 |  |  |
| **Likelihood Ratio** | .174 | 1 | .677 |  |  |
| **Fisher's Exact Test** |  |  |  | .699 | .387 |
| **Linear-by-Linear Association** | .173 | 1 | .677 |  |  |
| **N of Valid Cases** | 321 |  |  |  |  |

a Computed only for a 2x2 table

b 0 cells (.0%) have expected count less than 5. The minimum expected count is 38.39.

**9. Table 6**

**Oliguria (excluding patients with established oliguric renal failure), with diuresis < 0.5 ml/kg estimated body weight/hr**

**Randomization * oliguria cross tabulation**

|  | |  | **Oliguria** | | **Total** |
| --- | --- | --- | --- | --- | --- |
|  | |  | **0** | **1** | **0** |
| **Randomization** | 1 | Count | 122 | 43 | 165 |
|  |  | % within randomization | 73.9% | 26.1% | 100.0% |
|  |  | % within oliguria | 50.6% | 53.8% | 51.4% |
|  |  | % of total | 38.0% | 13.4% | 51.4% |
|  | 2 | Count | 119 | 37 | 156 |
|  |  | % within randomization | 76.3% | 23.7% | 100.0% |
|  |  | % within oliguria | 49.4% | 46.3% | 48.6% |
|  |  | % of total | 37.1% | 11.5% | 48.6% |
| **Total** | | Count | 241 | 80 | 321 |
|  | | % within randomization | 75.1% | 24.9% | 100.0% |
|  | | % within oliguria | 100.0% | 100.0% | 100.0% |
|  | | % of total | 75.1% | 24.9% | 100.0% |

**Chi-Square Tests**

|  | **Value** | **df** | **Asymp. Sig. (2-sided)** | **Exact Sig. (2-sided)** | **Exact Sig. (1-sided)** |
| --- | --- | --- | --- | --- | --- |
| **Pearson Chi-Square** | .235(b) | 1 | .628 |  |  |
| **Continuity Correction(a)** | .127 | 1 | .722 |  |  |
| **Likelihood Ratio** | .235 | 1 | .628 |  |  |
| **Fisher's Exact Test** |  |  |  | .699 | .361 |
| **Linear-by-Linear Association** | .234 | 1 | .628 |  |  |
| **N of Valid Cases** | 321 |  |  |  |  |

a Computed only for a 2x2 table

b 0 cells (·0%) have expected count less than 5. The minimum expected count is 38·88.

**10. Table 7**

**Elevated blood lactate (> 50% above upper normal limit) and clinical suspicion of hypoperfusion**

**Randomization * increased lactate cross tabulation**

|  | |  | **Increased lactate** | | **Total** |
| --- | --- | --- | --- | --- | --- |
|  | |  | **0** | **1** | **0** |
| **Randomization** | 1 | Count | 148 | 17 | 165 |
|  |  | % within randomization | 89.7% | 10.3% | 100.0% |
|  |  | % within increased lactate | 51.2% | 53.1% | 51.4% |
|  |  | % of total | 46.1% | 5.3% | 51.4% |
|  | 2 | Count | 141 | 15 | 156 |
|  |  | % within randomization | 90.4% | 9.6% | 100.0% |
|  |  | % within increased lactate | 48.8% | 46.9% | 48.6% |
|  |  | % of total | 43.9% | 4.7% | 48.6% |
| **Total** | | Count | 289 | 32 | 321 |
|  | | % within randomization | 90.0% | 10.0% | 100.0% |
|  | | % within increased lactate | 100.0% | 100.0% | 100.0% |
|  | | % of total | 90.0% | 10.0% | 100.0% |

**Chi-Square Tests**

|  | **Value** | **df** | **Asymp. Sig. (2-sided)** | **Exact Sig. (2-sided)** | **Exact Sig. (1-sided)** |
| --- | --- | --- | --- | --- | --- |
| **Pearson Chi-Square** | .042(b) | 1 | .837 |  |  |
| **Continuity Correction(a)** | .000 | 1 | .985 |  |  |
| **Likelihood Ratio** | .042 | 1 | .837 |  |  |
| **Fisher's Exact Test** |  |  |  | .855 | .493 |
| **Linear-by-Linear Association** | .042 | 1 | .837 |  |  |
| **N of Valid Cases** | 321 |  |  |  |  |

a Computed only for a 2x2 table

b 0 cells (.0%) have expected count less than 5. The minimum expected count is 15.55.

**11. Table 8**

**Acute alteration of mental status related to hemodynamic alterations**

**Randomization * altered ment cross tabulation**

|  | |  | **altered ment** | | **Total** |
| --- | --- | --- | --- | --- | --- |
|  | |  | **0** | **1** | 0 |
| **Randomization** | 1 | Count | 162 | 3 | 165 |
|  |  | % within randomization | 98.2% | 1.8% | 100.0% |
|  |  | % within altered ment | 52.1% | 30.0% | 51.4% |
|  |  | % of total | 50.5% | .9% | 51.4% |
|  | 2 | Count | 149 | 7 | 156 |
|  |  | % within randomization | 95.5% | 4.5% | 100·0% |
|  |  | % within altered ment | 47.9% | 70.0% | 48.6% |
|  |  | % of total | 46.4% | 2.2% | 48.6% |
| **Total** | | Count | 311 | 10 | 321 |
|  | | % within randomization | 96.9% | 3.1% | 100.0% |
|  | | % within altered ment | 100.0% | 100.0% | 100.0% |
|  | | % of total | 96.9% | 3.1% | 100.0% |

**Chi-Square Tests**

|  | **Value** | **df** | **Asymp. Sig. (2-sided)** | **Exact Sig. (2-sided)** | **Exact Sig. (1-sided)** |
| --- | --- | --- | --- | --- | --- |
| **Pearson Chi-Square** | 1.893(b) | 1 | .169 |  |  |
| **Continuity Correction(a)** | 1.112 | 1 | .292 |  |  |
| **Likelihood Ratio** | 1.937 | 1 | .164 |  |  |
| **Fisher's Exact Test** |  |  |  | .208 | .146 |
| **Linear-by-Linear Association** | 1.887 | 1 | .170 |  |  |
| **N of Valid Cases** | 321 |  |  |  |  |

a Computed only for a 2x2 table

b 1 cells (25.0%) have expected count less than 5. The minimum expected count is 4.86.

**12. Results**

**Additional results**

The following variables were evaluated for the analysis of reaching stability at 6 hrs: randomization, presence of each of the individual criteria of hemodynamic instability at baseline, and mechanical ventilation at baseline. The following variables were different between those, who achieved vs. did not achieve stability at 6 hrs, at p level of less than 0.10, and were subsequently included in a logistic regression analysis for predictors of hemodynamic status (stable/unstable) at 6 hrs:

Clinically relevant hypotension p=0.019

Acute symptomatic decrease of blood pressure p=0.013

Acute reduction in urinary output related to blood pressure decrease p=0.078

Oliguria p=0.022

Increased blood lactate p=0.001

In the logistic regression analysis, only acute symptomatic decrease in blood pressure at baseline (p=0.050) and increased blood lactate at baseline (p=0.002) were significant predictors of instability at 6hrs.

The mean (SD) SOFA sum score (without central nervous system score due to frequent use of sedation) and the SAPS II score for patients achieving vs. not achieving stability within 6 hrs was 4.6 (2.6) vs. 6.5 (3.1) and 41.4 (15.3) and 50.5 (16.1) for SOFA (p=0.000) and SAPS II (p=0.000), respectively. If these variables were added in the previous logistic regression analysis, then increased lactate at baseline (p=0.007), SOFA score (p=0.002), and SAPS II (p=0.025) were significant predictors of instability at 6 hrs.

The following variables were evaluated for the analysis of differences between hospital survivors and non-survivors: randomization, presence of each of the individual criteria of hemodynamic instability at baseline and at 6 hrs, mechanical ventilation at baseline and at 6 hrs, stability at 6 hrs and 24 hrs. The following variables were different between the survivors and non-survivors at p level of less than 0.10, and were subsequently included in a logistic regression analysis for predictors of hospital outcome:

Signs of cerebral hypoperfusion at baseline; p=0.06

Signs of renal hypoperfusion at baseline; p=0.01

Oliguria at baseline; p=0.000

Increased lactate at baseline; p=0.000

Decreased venous filling at 6 hrs; p=0.07

Oliguria at 6 hrs; p=0.08

Increased lactate at 6 hrs; p=0.03

Stability achieved at 6 hrs; p=0.04

Stability achieved at 24 hrs; p=0.000

Time to achieve stability; p=0.003

When only the baseline variables were included in the model, oliguria (p=0.000) and increased lactate (p=0.002) were significant predictors of hospital mortality. When only the 6-hr variables were included in the model, none of them was predictive of hospital mortality. When all the variables were included, only oliguria (p=0.000) and increased lactate (p=0.014) at baseline were significant predictors of hospital mortality.

Since oliguria and increased blood lactate may be related to other reasons than hemodynamic instability, we further analyzed the patients with oliguria or increased lactate at baseline in more detail. Of patients with oliguria at baseline, 91% had other instability criteria as well (median 2, interquartile range 1-2). Of patients with increased lactate at baseline, 87% had other instability criteria as well (median 2, interquartile range 1-2). Patients with oliguria alone (MICO n=7, control n=4) and increased lactate alone (MICO=7, control=7) at baseline were quite evenly distributed between the study groups. Furthermore. almost all of the patients with either oliguria or increased lactate as the single instability criterion at baseline had further instability criteria within the first 6 hrs despite ongoing treatment (3 patients had increased lactate and 1 patient oliguria as the sole instability criterion throughout the first 6 hrs; all in the MICO group).

SD = standard deviation. SOFA = sequential organ failure assessment. SAPS II = simplified acute physiology score II. MICO = minimally invasive cardiac output.
